# Mothers’ Experiences and Challenges Raising a Child with Autism Spectrum Disorder: A Qualitative Study

**DOI:** 10.3390/brainsci11030309

**Published:** 2021-03-02

**Authors:** Dimitrios Papadopoulos

**Affiliations:** 1 Department of Psychology, Gallos University Campus, University of Crete, 74100 Rethymno, Greece; d.papadopoulos@uoc.gr; 2 Child and Adolescent Mental Health Service, Panhellenic Association of Mental Health for Children and Adults, 17671 Athens, Greece

**Keywords:** autism spectrum disorder, child, mother, family, burden, childrearing

## Abstract

Although the study of autism is burgeoning with important implications both for public health and society, there is little research exploring the experiences of raising a child with autism spectrum disorder (ASD) from the maternal perspective. The aim of this study was to investigate the lived experiences of mothers of children with ASD in Greece. Nine mothers of children with ASD were recruited and engaged in semistructured interviews. Transcripts of the interviews were analyzed using interpretative phenomenological analysis. Three interconnected themes were identified in the analysis: (a) emotional burden, (b) family burden, and (c) social burden. A key finding in the themes was the sense of burden, distress, and vulnerability experienced by the mothers. The findings provide valuable understanding of the experiences of mothers raising children with ASD in one of Europe’s medium-income countries. Further, results can be used by researchers, clinicians, mental health providers, and policy makers to address the unique needs of families caring for and supporting children with ASD.

## 1. Introduction

ASD is a lifelong neurodevelopmental disability characterized by persistent and pervasive impairments in social understanding and communication, poor adaptive functioning, and the presence of restricted or repetitive behaviors and interests [1]. Current epidemiological studies have revealed an increased prevalence of ASD over the previous two decades [2]; ASD has been diagnosed more often in boys than in girls, with a sex ratio of 3 (boys): 1 (girls) [3]. According to estimates from the CDC’s Autism and Developmental Disabilities Network, the current prevalence is 1 in every 54 children in the United States [4], while in recent years, the prevalence in Europe has increased rapidly, due to an increased awareness of autism and hence an increased likelihood of the condition being diagnosed [5]. Data suggest that the estimated prevalence of autism diagnosis in Greece approximates 1.15% of school-age children born in 2008 and 2009 (1.83% of males and 0.44% of females) [6], which is close to the value for Europe overall [7] and internationally [8].

Parenting a child with ASD is a stressful and challenging experience [9,10], particularly in countries where numerous support services are limited. The literature indicates that caregivers of children with ASD often experience impaired mental health, including anxiety and depression [11,12], a poorer quality of life and wellbeing, and higher levels of stress as compared to caregivers of typically developing children [13], and caregivers of children with other childhood disorders such as Down’s syndrome and/or ADHD [14]. Previous studies revealed that mothers of children with ASD reported elevated psychological distress and caregiving burden [15], health-related problems [16], lower levels of resilience [17] and problems in various areas of family life, including marital and sibling relationships and family socialization [18]. Fairthorne et al. [16] conducted a study in Western Australia from 1983 to 2005, including mothers with live-born children with autism. They found that participants were not only distressed, but also were vulnerable to severe medical conditions such as cancer and had an increased risk of early death. More recently, Gobrial [19] conducted a qualitative study of Egyptian mothers of children with ASD. The findings indicated that the diagnosis of autism had a negative effect on the mothers’ social life and mental well-being; caring for a child with autism increased stigmatization of both mothers and their children. In addition, research suggests that financial concerns, such as economic burden and the need for extra income to cover the lifelong high cost of ASD-related special education and treatment, are important factors that contribute to increased parental fatigue, specifically for low-income households [20].

A body of research has highlighted that stressors of parents of children with autism include the severity of autism symptoms and the level of disability [21,22], such as the intelligence quotient (IQ) of the child [23]. Thus, it appears that a child with a low level of cognitive development and functional abilities may increase the impact of parental stress due to the child’s propensity for long-term dependency. Additionally, 92% of children with autism exhibit psychiatric comorbidities, including attention-deficit/hyperactivity disorder, oppositional defiant disorder, and anxiety disorder [24], as well as behavioral problems including noncomplianceand defiance [25]; these issues have a direct and positive effect on mental tiredness among mothers and fathers [26]. This effect seems to be greater as children age and these problems typically occur and are recognized by teachers and caregivers. In such cases, parents may experience elevated stress because they face challenges in managing the child’s comorbidity-related deficits [27].

Similarly, research has shown that a lack of perceived social support among mothers of children with autism is the most significant indicator of the development of mental health problems [28] and has a negative impact on parental stress and on family socialization [29]. Sanders and Morgan [30] found that an absence of social support services contributes to parents facing more challenges to their parenting skills associated with the child’s characteristics, which in turn exacerbates parental stress and depressive symptoms. Indeed, the child’s characteristics generally influence the mother’s decision to seek formal social support, such as professional-based assistance and respite services, to help parents address the child’s problems and reduce the social stigma faced by the child [31]. From a different perspective, strong protective factors against maternal stress include the use of both formal and informal sources of social support, such as professional guidance, groups of friends, extended family members, groups of parents with similar concerns, and online communities (e.g., Facebook) [32]; these sources help parents to maintain a positive mood and emotional wellbeing [33] and increase the size of their support network, thereby aiding successful coping with higher levels of distress [34,35]. Al-Kandari et al. [36] and Cetinbakis, Bastug and Ozel-Kizil [37] highlighted a significant relationship between receiving family and social support and the ability of mothers to enjoy life and be optimistic, which are associated with higher levels of positive maternal outcomes and decreased expression of negative emotions [38]. Other studies suggest that social support focused on the family is a crucial factor that contributes to family adaptation and resilience [39], which in turn increases the family’s sense of control over life events [40].

Previous studies [41,42] indicated that cultural context may play a role in the stress experienced by parents of children with ASD. In addition, parental beliefs about ASD can be negative (e.g., the child is “defective and the impairment needs to be fixed”) or positive (e.g., this is a challenge for a parent to become a better person), and such appraisals of the concept of disability can be culturally determined [43]. In Greece mothers’ experiences of caring for a child with ASD may be affected by contextual and culture-specific factors in ways that have not yet been fully examined. First, children with developmental disabilities and their families face a lack of access to equal opportunities in education and health [44]. This may happen because such services offered by the state are minimal and in general do not best meet the wholistic needs of children and their families. Efforts have been made in recent years to comprehensively improve the Greek education and social care system, with the aim of including children with disabilities, but progress remains poor relative to the average progress achieved in the European Union [44]. Second, mothers in Greece are more likely to take on a family caregiving role compared to fathers, who are less involved in parenting their children and mainly tend to adopt disciplinary roles [45]. This is potentially a significant environmental factor that could contribute to Greek mothers’ relatively greater burden. A previous qualitative study from Greece [46] revealed an interesting finding: fathers were not allowed by mothers to undertake “female activities,” which may reflect confusion regarding beliefs with respect to gender roles in family responsibilities. Third, over the past decade, Greek society in general has faced considerably higher socio-economic challenges than Europe as a whole, which have led to greater barriers to accessing and using healthcare services for the most vulnerable populations, including people with disabilities and their families [47].

Although autism studies worldwide have recently gained significant recognition, the experience of raising a child with autism in Greece is seriously underrepresented and unexplored. To the author’s knowledge, only one published qualitative study has considered the experiences of mothers of children with ASD in Greece: Loukisas and Papoudi [48] illustrated that a sample of five mothers reported elevated levels of anxiety, depression, and distress; in addition, the mothers faced social stigma. Other studies in Greece have considered ASD’s psychosocial impact on families associated with parental needs and concerns. A cross-sectional study by Ntre et al. [49] found that ASD has a significant impact on maternal wellbeing and family life, including family finances. Likewise, Kostantareas and Papageorgiou [50] found that maternal stress is predicted by the severity of the child’s ASD symptoms, activity level, and negative mood.

Therefore, the current qualitative study considered the lived experiences and challenges of parenting among mothers of children with autism in Greece. This study provides insight into the experiences of mothers caring for children with ASD, but also provides knowledge that can be used to raise public awareness of the unmet maternal psychological needs associated with having a child with ASD and provides empirical information to support researchers and policy makers in developing family focused interventions and education programs for autism in Greece.

## 2. Materials and Methods

To understand the experiences of mothers of children with ASD, this study employed an exploratory qualitative method. An interpretative phenomenological analysis (IPA) was used to evaluate in-depth interviews, as this approach allows participants to articulate their experiences in great detail and permits better understanding of what a group that shares similar concerns feels and believes about their own worldview [51].

### 2.1. Participants

A convenience sample was recruited of nine mothers living in the metropolitan area of Athens whose children aged 5–9 years old had been referred to a child and adolescent mental health service (CAMHS). All children had received a diagnosis of autism spectrum disorder according to the DSM-5 criteria [1] by a pediatric mental health multidisciplinary team. To be included in the study, mothers were required to speak the Greek language, currently live with their children, and have maternal responsibilities.

Of the nine participants, eight were Greek, one was Albanian; seven were currently married, and two were divorced. Three mothers reported a history of depression. Most (*n* = 7) participants had a son with ASD, while two mothers had a daughter with ASD. Almost half (*n* = 5) of the children had comorbid behavioral disorders. Two-thirds of the children (6 out of 9) attended regular classes in public elementary schools under the care and supervision of special teachers, whereas the other children (3 out of 9) attended an integration classroom in a public or private kindergarten. All participants had earned high school diplomas, and four of them held an undergraduate diploma or higher. A summary of the participants’ sociodemographic characteristics is provided in Table 1.

### 2.2. Data Collection Procedure

Interview data were collected using a face-to-face, semi-structured, in-depth qualitative interview that was conducted by the author, following a sequence of eight open-ended questions. Semistructured interviews gave the parents the chance to express in detail their personal experience of raising a child with ASD. Interviews were conducted in accordance with the overall principles established by Smith and Osborn [52] for use in IPA. The interviews were conducted either in an office at the CAMHS or in the participants’ homes. All interviews lasted between 45 and 90 min.

The interview protocol was designed to elicit in-depth mothers’ perspectives (i.e., thoughts, feelings, and experiences) through the course of the child’s disability in the present and in the past. Mothers were asked to describe, in their own words, how having and raising a child with autism affected various aspects of their personal, social, and family life, and how this experience affected their needs. The interview started with a very broad question: “Tell me about your experience as a mother raising a child with autism in Greece”; examples of specific questions from the interview protocol include “How has your child’s autism affected your life most?”, “Could you describe your relationship with your child and family members?”, and “Looking forward, what worries do you have about your child’s future?” Given the responses of the participants, extra probes were used to collect additional information and data regarding the themes that the mothers perceived as most relevant. All mothers received the questions in the same order.

To ensure the quality and accuracy of the data, interviews were audio recorded and subsequently transcribed verbatim by the author and then checked by two independent PhD-level psychologists. All participants completed a sociodemographic questionnaire after the interview was conducted. The questionnaire asked for the mother’s age, level of schooling, religious beliefs, as well as the child’s age and diagnosis.

### 2.3. Ethics

Ethical approval was obtained from the “Panhellenic Association of Mental Health for Children and Adults” ethics committee. The ethical guidelines followed were consistent with American Psychological Association research ethics recommendations, as in Greece, there is a lack of specific rules by which to conduct qualitative studies [53]. For example, mothers were informed that their participation was voluntary and that they could withdraw from the interview process at any time. All participants were given consent forms prior to the start of the interview, which indicated that socio-demographic characteristics might be published, as might participants’ direct quotes. All mothers were ensured that their personal information would be modified to protect confidentiality and anonymity, using pseudonyms.

### 2.4. Data Analysis and Validity

IPA was used to analyze the transcripts, to “give voice” to the participants’ essential life viewpoints [54]. Thus, an in-depth inductive qualitative analysis was conducted to uncover the mothers’ unique perspectives with regard to raising a child with autism, through a detailed examination of their personal perceptions and lived experiences.

Transcripts were coded and analyzed by the author following a step-by-step transcript analysis according to the IPA method [51,52], which entailed organizing, coding, integrating, and interpreting the data [55]. During the text review, the author focused more on the subjective point of view of the participants than on factual data. First, the author read and reread each participant’s transcript separately in order to familiarize himself with the data. This process was repeated several times for all transcripts, enabling the author to obtain a holistic overview of the participants’ thoughts and feelings through their stories. Moreover, at this phase, the author recorded his observations and comments [52].

In the next phase, the author performed a line-by-line coding of each interview transcript. Important descriptive, linguistic, and conceptual themes for each transcript emerged by focusing on connections across the themes with respect to each single narrative and then across the narratives.

The analysis was subsequently reviewed and developed further by the author and two independent PhD-level psychologists, whose insights were incorporated into the analysis. After iterative review and discussion phases, the analysis of each interview resulted in overall agreement regarding the final analysis and written report. The findings are described with relevant narratives from the mothers, which were translated verbatim from Greek into English to highlight the original narratives.

To establish the credibility and validity of the results, the interviews were independently coded by the author and two independent PhD-level psychologists; codes were discussed through researcher-supervisor debriefing to enable triangulation, which both enriched the analysis and served as a quality control procedure. Moreover, to further ensure trustworthiness, reflexive discussions were conducted to explore whether the researcher’s previous beliefs about maternal experiences of raising a child with ASD may have influenced his bias, and therefore the results of the analysis.

## 3. Results

The analysis revealed three major themes concerning the maternal burden of raising a child with ASD: emotional burden, family burden, and social burden. For each of the superordinate themes, a set of subthemes also emerged (see Table 2).

### 3.1. Emotional Burden

The theme of emotional burden was identified by all participants when they were asked about the overall impact of raising a child with autism. Most of the mothers became tearful during interviews when describing their feelings and reported overwhelming emotional reactions to their child’s diagnosis, daily struggles related to the child’s caregiving, and thoughts about the child’s development and future.

#### 3.1.1. Emotional Reaction to the Child’s Diagnosis

Some participants reported that various negative emotional reactions occurred following the child’s autism diagnosis. One mother described that when she learned of her son’s diagnosis, she entered a phase of depression and felt distressed.

“After the initial diagnosis I couldn’t get out of bed and face the day, I felt mentally exhausted and depressed”.

(Maria)

Another mother (Anna) reported that “I felt disconnected from myself. I felt always tired and wanting to stay in bed, and I did not control my child’s behavior.” Furthermore, most of the mothers reported a sense of guilt and shame after the diagnosis of ASD, which in turn resulted in their distress and emotional fatigue.

“I believed that I did something wrong; I blamed myself because my child was not normal; I wondered if this condition was caused by my behavior as a mother. I was distressed for a long time after the diagnosis, and I cried every day before sleeping”.

(Eva)

“I had the impression that I was at fault for my child’s disorder; am I the source of the problem?”.

(Lia)

Although some mothers felt guilt, one mother appeared to appreciate that she had not caused her child’s autism.

“Because I read many books and I search the scientific data, I’m aware that autism was not caused by my behavior as a mother”.

(Dina)

#### 3.1.2. Feelings of Helplessness and Frustration

All mothers reported feelings of helplessness regarding the challenging daily childcare demands.

“My son needs much attention and focus 24 h per day in order to control his behavioral difficulties. You know, it’s very frustrating to have to treat his needs every day.Sometimes, I feel I do not love this child enough, and this thought makes me feel a sense of guilt”.

(Katerina)

Moreover, all mothers in this sample described feeling distress linked to not knowing what they could do, or who they could rely on, to help their child. This sense of uncertainty emerged as a sense of frustration. Ria, for example, described the following:

I am lonely, I do not have a partner, and it’s hard to be a single mother when you have a child with special needs; commonly, I feel fear and uncertainty in managing my child’s demands; not knowing how to help my son when he begins repetitive behavior. I’m confused.”

Anna often felt “overall empty, empty physically and mentally” particularly when she could not effectively cope with her child’s behavior, whereas Nancy reported feeling “burned out and feeling empty of available energy” regarding her son’s difficulty in making transitions and in self-managing unexpected changes in his daily routine. Dina described her frustration related to trying to reduce her child’s disruptive behavior, which she felt incapable of doing.

“I think the feeling of powerlessness is the worst, you want to do the best for your child, but you realize that you are not able to offer help”.

(Dina)

One mother described feeling frustrated as part of the process of raising a child with autism.

“Living with a child with autism is totally different from what the textbooks write. You can actually be frustrated easily when you hold full responsibilities of raising a child with special needs”.

(Lia)

#### 3.1.3. Child’s Future

Mothers reported constant worry about the long-term impact of autism on the child’s future. Indeed, the transition from childhood to adolescence and adulthood generated anxiety for all mothers involved, regardless of the child’s age. Moreover, uncertainty about the child’s education and, subsequently, job opportunities, living conditions, and the ability to adapt successfully to adulthood contributed to an intense sense of worry. The essence of her future-oriented fears was reported by the mother of a 9-year-old girl with autism.

“You start worrying what’s going to happen to her long term. I’m thinking 10–20 years from now how my child will fit to the environment, will she have the ability to create romantic relationships? Will she be able to study in college to earn money and live independently?”.

(Sofia)

Nancy worried and felt “uncertainty regarding her son’s transition from kindergarten to elementary school.” In addition, some mothers referred to the possible trajectory and consequences of autism for the child’s future mental health state.

“I wouldn’t imagine (my child) to be diagnosed with something long-term either, bi-polar or sort of (trails off)”.

(Ria)

At this point in the interview, Ria seemed unable to find the appropriate words to express what other long-term concerns could be, indicative of her anxiety about the future.

“It’s not just the concern for the poor school outcomes and related-barriers to find a job, but it’s the concern of development psychiatric comorbidities in the future, it’s just an overwhelming thought.”.

(Maria)

### 3.2. Family Burden

#### 3.2.1. Relationship with Spouse

The second superordinate theme captured by the mothers’ experiences was that autism had caused a change in family life, as the whole familial ecology had to adapt to a new reality; namely that the child needed special parental treatment and long-term care. All of the mothers in this study noticed that after the child’s diagnosis, their relationship with their spouse changed. Most mothers in this sample felt as though they had neglected the needs of their spouse, leading to emotional distance and deterioration compared to previously healthy connections. More specifically, Anna reported:

“There are times that you feel disconnected from your spouse, and the child’s needs are the priority; the husband is sort of second, third down the line if there are other kids.”

“I feel so tired and exhausted from the child’s and house responsibilities; therefore, at night, I feel unable even to kiss and hug my husband, in addition to spending more romantic time with him”.

(Katerina)

In addition, the mothers identified a marked difference in their spouse’s behavior following the diagnosis of autism. Typically, the mothers felt that their spouses had “shut down” (Maria) and that spouses more often expressed “aggressiveness and emotional distance” (Lia). For several mothers, this was a negative change that affected their relationship and communication; hence, they experienced “a sense of loss” (Alexia). Another mother described problems in the spousal relationship.

“I judged and criticized my husband because he had a different perspective on the child’s treatment; we fought a lot, yelled and screamed, we just didn’t get along the child’s condition affected our intimate life and finally we divorced”.

(Dina)

Only one mother (Eva) reported that hope for relief and positivity in family life developed when her child at times displayed a positive behavior change and improved his adjustment to school.

#### 3.2.2. Relationships with Siblings

Mothers described in predominantly negative terms how having a child with ASD influenced their relationship with their other typically developing children. This was frequently indicated by a reduction in the time the other sibling and mother spent together in shared activities.

“My son has become unfortunately the center of the family life, which I don’t like at all. However, me and my husband both have limited available time and energy to focus our attention on the needs of the typically developing sibling. This often leads to feelings of guilt and inappropriate parenting” .

(Sofia)

Additionally, Dina reported “Aris feels neglected and experiences negative reactions towards me as mother.”

“One day after a disagreement with my first-born child, the other child said to me and my husband you do not love me enough; the center of your lives is caring for my sibling’s special needs’, it was overwhelming to hear”.

(Lia)

#### 3.2.3. Family Finances

Financial strain emerged as a major concern; families in this study were forced to make significant lifestyle adjustments. This was caused by various factors, such as poor insurance coverage for needed services and higher costs for intense interventions (i.e., speech and occupational therapy, psychotherapy). Two mothers stated that they had to stop their child’s speech therapy and behavior therapy due to financial difficulties.

“These therapies are too expensive and exceeded the family financial resources, and only a small amount of the total cost is covered by the public insurance”.

(Maria)

In addition, many mothers reported that fathers faced a shift in career paths to fulfill the everyday requirements of childcare.

“My husband left his career and works as taxi driver to earn more money, and even that is not enough to save some money for his future”.

(Lia)

Other mothers were forced to stop working, and others made career-related sacrifices, such as seeking an employer more understanding of the demands of raising a child with ASD.

“I have had to put on hold many things. I stopped working and left my PhD studies to give my full attention to my child’s care”.

(Eva)

### 3.3. Social Burden

#### 3.3.1. Stigma of Autism

Most of the mothers reported that they had experienced autism stigma and they were forced to remain at home more frequently, rather than going out. Reasons for stigma included negative social stereotypes regarding mental illness and disability.

“You understand other people looking at you differently like we are coming from a different planet. I think society does not accept both me and my child stigma has changed my inner self; I hide from others that my child has autism and receives specific treatments”.

(Nancy)

The impact of autism stigma in this description is powerful and highlights the social pressure experienced by these mothers. According to one, the impact of living socially excluded is “mentally and physically exhausting” (Katerina).

Moreover, seven out of nine mothers described that their children were treated differently by typically developing children in school, as well as by teachers and other parents.

“In school other children do not want to either play with my child or to speak with him. They believe he is *crazy* and call him not by his name but as *hey autistic*”.

(Ria)

“Some mothers may take away their child from my child they are afraid of the stigma arising from playing with an autistic child”.

(Alexia)

Furthermore, several mothers described feeling embarrassed about their child’s behavior when the child met relatives, because “other people believe that autism is associated with bad parenting and a mother’s inability to control her child” (Dina).

#### 3.3.2. Social Life

Mothers reported a significant decrease in the quantity and quality of their social ties and relationships. Factors that helped establish this situation were a fear of stigmatization by others, a feeling that the mothers could no longer relate to old friends since the latter did not have similar concerns, limited time to spend socially, and an inability to bring children to social gatherings due to behavioral concerns.

“I had close friends from college who don’t have a child with disability and I really like it very much. Now, I have more autism mommies. I get bored of communicating with autism mommies, but I feel comfortable, and they’re comfortable. To be honest, I miss my old life. I miss one of my best friends. However, when you have a child with autism, your whole life is affected [crying]”.

(Sofia)

Most participants reported a tendency to avoid meeting other families and relatives with typically developing children (Alexia), and they explained that this had a negative effect on the social life of the family and children (Lia). A mother reported:

“Of course, I avoid going to social events (i.e., birthdays, party, wedding); these events are not for a family who have a child with autism; there is a high risk of social judgment when the kid becomes nervous”.

(Maria)

Importantly, the two employed mothers were more likely to feel socially included compared to mothers without employment outside the home.

“I’m very lucky to have my job and career; I feel accepted at work and I can show a different aspect of my identity. I am not only the mother of a child with autism”.

(Nancy)

## 4. Discussion

This study used a semistructured interview to explore the mothers’ firsthand accounts of their experiences of raising a child with autism in Greece. IPA analysis resulted in three main themes that described the maternal perspective: emotional burden, family burden, and social burden. The mothers who participated in this study shared information that provided evidence of many emotional, familial, social, and financial challenges associated with raising and supporting a child with ASD.

The first major theme to emerge from this study involved the emotional burden of the child’s diagnosis, feelings of frustration, and worries for the child’s future. In this sample, all mothers were the primary caregiver and perceived their major role as providing and coordinating care for the child in combination with other daily activities, including housekeeping and caring for the other family members’ needs. These numerous caregiving duties negatively impacted maternal mood and well-being, and created feelings of helplessness and powerlessness that increased pessimism regarding the future of their affected child. The current findings support previous studies that found an increased emotional burden associated with raising a child with autism, including increased levels of parenting stress, worry, and guilt among mothers, as well as feelings of being overwhelmed [56,57]. The mothers’ observations regarding the emotional impact of the child’s diagnosis concur with wider evidence from qualitative studies of parents of children with a range of developmental and mental health disorders who commonly expressed a desire not only to explain the individual experience, but further to search for the answer to an existential question, namely “why did this happen to us?” [58]. The idea that mothers are readily distressed echoes previous qualitative work, which indicated that parents of children with ASD experience distress and emotional turmoil [59] in relation to their child’s potentially dangerous and unpredictable behavior and functional dependency.

Interestingly, mothers in this study reported a lack of knowledge about how to effectively manage their child’s difficulties and deficits, which may have a negative effect on the care provided to the child. This finding has been highlighted in existing research, which has indicated a bidirectional relationship between the burden of care and the ability of parents to effectively cope with their child’s behavior and implement appropriate parenting practices [60,61]. Indeed, some parents of disabled children may lack an adequate understanding of their child’s developmental needs and behavioral demands while others may not use positive skills when developing parent-child interactions and rewarding the appropriate behavior of the child [62]. In contrast, parents with positive experiences of parenting their children tend to perceive themselves as effective parents more often. In addition, mothers in this study described elevated maternal worries about the child’s future and the long-term care of ASD; in particular, they reported concerns regarding potential psychiatric comorbidities, consistent with literature indicating symptoms of anxiety [63,64] and depression [65] among individuals with autism.

The second theme identified in the current study captured the mothers’ experiences of a significant change in the entire family system, including an increase in problems with spouses and sibling relationships, and economic impacts. Notably, mothers prioritized the needs of their children with ASD and focused on coping with the elevated childcare challenges, which resulted in changing family dynamics. These concerns expressed by the mothers’ echoed those in previous studies with parents of children with disabilities, which identified a breakdown in the relationship between family members and insufficient available time and energy for mothers to commit to their spouse or the siblings of the child with ASD [57]. Indeed, research has revealed a higher rate of divorce for parents of children with ASD than in families of children without disabilities [66,67]. Moreover, even for parents who remain married, raising a child with autism is associated with lower marital satisfaction compared to that of married parents of typically developing children [68]. Therefore, the impact of ASD on family life and relationships lends some support to family focused approaches, which suggest that a family member with autism exerts pervasive and bidirectional influences on the family system [69]. This can lead to increased expression of negative emotions (i.e., expression of hostility, rejection, and/or excessive emotional involvement in relationships between the person with autism and his/her family), which creates family stress and thereby plays a role in the maintenance and treatment of the ASD [70].

Further, mothers expressed financial strain arising from the overall cost of care, related to both current and future treatment expenses. These findings are consistent with several studies, suggesting that having a child with autism is associated with a reduction in family income, while other parents need to work extra hours or change jobs to cover the high cost of their child’s special education and medication [49,71]. According to Montes and Halterman [72], childcare concerns affected the employment decisions of parents in the United States, and the estimated loss of annual expenses related to raising a child with autism was $6200 or 14% of their recorded income. The financial impact on Chinese families of a child with autism was 16 times higher than of a typically developing child [73]. Indeed, this phenomenon is particularly clear in Greece, where there is a lack of public mental health facilities for children with disabilities, and parents are required to pay themselves for specific treatments offered in private clinics where the monthly costs range from €401 to €1000. This may represent a source of stress to parents. Previous studies from Greece [6,74] illustrated that due the severe economic crisis of the preceding decade, families with low socioeconomic status have limited access to healthcare services. In addition, financial austerity significantly negatively affected the planning and development of the necessary resources and services in special education and mental health for individuals with ASD. These effects have impeded the positive development and psychosocial wellbeing of disabled people, and produced long-term disadvantages.

The final theme described the mothers’ social burden with respect to the stigma and the overall impact of ASD on their social life. Mothers in this study reported considerable social impact, including increased isolation from friends and extended family, since they avoided engaging in social activities due to elevated childcare responsibilities and worries regarding their child’s potential behavioral outbursts in public, consistent with previous research [75,76]. Mothers expressed concerns regarding the stigma of autism, which reflected the social stereotypes and discrimination that are still present in modern Greek society and are typically proliferated in mass media. Indeed, the social model of disability supports the idea that disability is a social disadvantage that limits and restricts the individual’s capabilities, while society tend to focus more on the negative consequences of the disability [77]. Additionally, mothers described predominantly experiences of autism stigma, including negative reactions and attitudes of others regarding their child’s unique characteristics. This is an important finding because research has found autism-related stigma to be associated with increased mental health problems among caregivers [78,79]. Further, stigma undermines the mothers’ confidence in their parenting skills and negatively impacts their self-self-esteem [80]. Consistent with the current findings, a qualitative study conducted by Loykisas and Papoudi [48] in Greece showed that caregivers experienced autism-related stigma arising from rejection within their environment since their children were characterized as “abnormal”, and were perceived as less acceptable than typically developing children by other parents. Furthermore, interviews with Egyptian mothers of autistic children indicated that to prevent stigmatization, these mothers tended to keep their children at home [19]. This, in turn, affected the mothers’ social life, such that they became socially isolated.

The implications of this study are that greater understanding of maternal experiences and concerns can serve as a guide to develop and deliver family centered services directed toward helping parents to identify their unique needs regarding the challenges of parenting a child with autism [70]. Family-centered interventions can enhance parenting skills and promote the mothers’ ability to cope with stressful emotions and obstacles that may present a family burden and can increase parent-child attachment by promoting love and intimacy among family members. Given that the mothers in this study reported feelings of self-blame and guilt for their child’s autism, it may be important for mental health providers to proactively offer psychoeducation alongside CBT, to explain to parents of children with autism the nature and the developmental course of the disorder through the various life stages. This approach could also illustrate behavioral strategies in which mothers can model self-care for their children and help to minimize feelings of powerlessness in such children by highlighting useful practical knowledge [62,81,82]. On the cognitive level, CBT interventions teach strategies to enable parents to change their negative thoughts and irrational beliefs, which in turn can reduce parenting stress and increase mental well-being. In addition, family interventions must focus not only on issues related to the affected child and his/her relationship with other family members, but also on supporting and teaching positive communication skills to spouses, such as demonstrating appreciation of the other’s needs as a parent and partner and recognizing one’s challenges when parenting a child with special needs [69,82].

Fear of being stigmatized by the autism label and the language used to describe autism led mothers to avoid engaging in social activities, which negatively impacted their social life outside of the immediate family. Therefore, service providers treating and educating the child with autism must understand the value of social support to create opportunities for individuals with autism and their families to overcome social barriers and discrimination [83]. By providing a continuum of social support services, agencies can enable families to develop in supportive community networks that best meet their unique needs. For example, parent support groups are an effective formal service by which to increase the family’s ability to cope with various stressors [28,56]. Furthermore, parent groups may be effective at increasing the parents’ acceptance of their child’s disability following discussions with other parents who face similar experiences and concerns. A major disadvantage of these services is that parents need to actively search for them; persons unfamiliar with accessing adequate community resources, such as new immigrants, may not benefit from these community-based services [41]. From a social perspective, policy-makers need to establish preventive education programs aimed at improving public awareness and fostering people’s deeper understanding of the characteristics and life-long needs of individuals with ASD. Learning to accept neurodivergent people may help to increase positive societal attitudes toward autism and reduce the social stigma associated with autism that impedes the inclusion of individuals with ASD in education and in overall social life [83].

### Strengths and Limitations

This study has several strengths. IPA was used to analyze the interview narratives and to describe the significance of these mothers’ current experiences. Consistent with the IPA method, the mothers who participated in the study were selected and interviewed in consistent manner, shortly after the ASD diagnosis of their children. The rationale for this detailed investigation was focused on the centrality of the role played by mothers as caregivers for their sons or daughters and as facilitators of their children’s access to treatment services. Furthermore, the study was carried out in a routine child and adolescent mental health center, thereby providing good ecological validity.

Although this study presents evidence that supports the impact of raising a child with ASD from the maternal perspective, some important limitations must be considered. First, the nature of qualitative research is that it does not seek to be generalizable; therefore, the findings cannot be assumed to apply to other mothers within this population across various contexts [51]. In addition, recruitment from a wider geographical region may help future studies to sample the diverse motherhood experiences in Greece. However, as the data were collected using a flexible semistructured interview schedule and in-depth analysis was conducted, rich and detailed data were obtained regarding the experiences of these mothers. Second, this study did not reflect the lived experiences of having a child with autism from a paternal perspective. This is particularly important given that the involvement of fathers has been consistently linked with a significant contribution to their children’s positive development [84]. Therefore, further studies should focus on the role of the father in caring for a child with autism spectrum disorder to gain insight into the fathers’ perspectives on stress and coping resources [85]. In addition, research integrating full-family conversations may be helpful in assessing common emotions and experiences, as interactions among family members can present often unique and meaningful findings that could contribute to the development of successful family-based interventions. Finally, future research should focus on parental experiences of caring for a family member with autism not only during childhood but also at various other ages, including adolescence and young adulthood.

## 5. Conclusions

This study provided useful insights into the mothers’ lived experiences of raising and caring for a child with childhood autism in Greece. The results of the current study revealed that families parenting a child with ASD experience several consequences across many aspects ranging from emotional and family burden to social and financial burden. Mothers reported their children’s behavior and their parenting role as the primary source of their caregiving burden and stress, with intense feelings of autism stigma perceived from their community. Interestingly, these issues had a considerable and broad impact on their own lives. Further research in broader and representative samples including mothers and fathers providing care to children with autism spectrum disorder is needed before these findings can be generalized.

## Figures and Tables

**Table 1:** Participants’ characteristics.

Mother	1	2	3	4	5	6	7	8	9
Mother’s age	38	31	34	43	37	33	30	39	42
Marital status	Divorced	Married	Married	Divorced	Married	Married	Married	Married	Married
Educational level	Undergraduate	Post high school	High school	Undergraduate	Postgraduate	Undergraduate	Post high school	High school	High school
Religious belief	Religious	Very religious	Atheist	Religious	Religious	Very religious	Religious	Very religious	Very religious
Number of children	1	2	3	2	2	1	1	2	2
Age of child with ASD	5 year, 6 months	5 years, 4 months	5 years, 8 months	9 years, 2 months	6 years, 9 months	8 years, 8 months	5 years, 7 months	7 years, 1 month	9 years, 6 months
Child’s gender	Male	Male	Male	Male	Male	Female	Male	Male	Female

**Table 2:** Superordinate and subordinate themes.

Superordinate Themes	Subordinate Themes
Emotional burden	Reactions to the child’s diagnosis
	Feelings of vulnerability and frustration
	Child’s future
Family burden	Relationship with spouse
	Relationship with siblings
	Family finances
Social burden	Stigma of autism
	Social life

## Data Availability

The qualitative data presented in this study are available on reasonable request from the corresponding author. The data are not publicly available due to the applicable data protection law in Greece (Law 4624/2019).
